# Clinical Importance of Clonal Hematopoiesis in Metastatic Gastrointestinal Tract Cancers

**DOI:** 10.1001/jamanetworkopen.2022.54221

**Published:** 2023-02-02

**Authors:** Bill H. Diplas, Ryan Ptashkin, Joanne F. Chou, Shalom Sabwa, Michael B. Foote, Benoit Rousseau, Guillem Argilés, James Robert White, Caitlin M. Stewart, Kelly Bolton, Sree B. Chalasani, Avni M. Desai, Zoe Goldberg, Ping Gu, Jia Li, Marina Shcherba, Alice Zervoudakis, Andrea Cercek, Rona Yaeger, Neil H. Segal, David H. Ilson, Geoffrey Y. Ku, Ahmet Zehir, Marinela Capanu, Yelena Y. Janjigian, Luis A. Diaz, Steven B. Maron

**Affiliations:** 1 Department of Radiation Oncology, Memorial Sloan Kettering Cancer Center, New York, New York; 2 Department of Pathology, Memorial Sloan Kettering Cancer Center, New York, New York; 3 Department of Epidemiology & Biostatistics, Memorial Sloan Kettering Cancer Center, New York, New York; 4 Department of Medicine, Memorial Sloan Kettering Cancer Center, New York, New York; 5 Resphera Biosciences LLC, Baltimore, Maryland; 6 Meyer Cancer Center, Weill Cornell Medicine, New York, New York; 7 New York Genome Center, New York, New York; 8 Department of Medicine, Washington University Medical School, St Louis, Missouri

## Abstract

**Importance:**

Clonal hematopoiesis (CH) has been associated with development of atherosclerosis and leukemia and worse survival among patients with cancer; however, the association with cancer therapy efficacy, in particular immune checkpoint blockade (ICB), and toxicity has not yet been established. Given the widespread use of ICB and the critical role hematopoietic stem cell–derived lymphocytes play in mediating antitumor responses, CH may be associated with therapeutic efficacy and hematologic toxicity.

**Objective:**

To determine the association between CH and outcomes, hematologic toxicity, and therapeutic efficacy in patients with metastatic gastrointestinal tract cancers being treated with systemic therapy, both in the first-line metastatic treatment setting and in ICB.

**Design, Setting, and Participants:**

This retrospective cohort study included 633 patients with stage IV colorectal (CRC) and esophagogastric (EGC) cancer who were treated with first-line chemotherapy and/or ICB at Memorial Sloan Kettering Cancer Center. Patients underwent matched tumor and peripheral blood DNA sequencing using the Memorial Sloan Kettering–Integrated Mutation Profiling of Actionable Cancer Targets next-generation sequencing assay between January 1, 2006, and December 31, 2020.

**Exposures:**

Clonal hematopoiesis–related genetic alterations were identified by next-generation sequencing of patients’ tumor and normal blood buffy coat samples, with a subset of these CH alterations annotated as likely putative drivers (CH-PD) based upon previously established criteria.

**Main Outcomes and Measures:**

Patients with CH and CH-PD in peripheral blood samples were identified, and these findings were correlated with survival outcomes (progression-free survival [PFS] and overall survival [OS]) during first-line chemotherapy and ICB, as well as baseline white blood cell levels and the need for granulocyte colony-stimulating factor (G-CSF) support.

**Results:**

Among the 633 patients included in the study (390 men [61.6%]; median age, 58 [IQR, 48-66] years), the median age was 52 (IQR, 45-63) years in the CRC group and 61 (IQR, 53-69) years in the EGC group. In the CRC group, 161 of 301 patients (53.5%) were men, compared with 229 of 332 patients (69.0%) in the EGC group. Overall, 62 patients (9.8%) were Asian, 45 (7.1%) were Black or African American, 482 (76.1%) were White, and 44 (7.0%) were of unknown race or ethnicity. Presence of CH was identified in 115 patients with EGC (34.6%) and 83 with CRC (27.6%), with approximately half of these patients harboring CH-PD (CRC group, 44 of 83 [53.0%]; EGC group, 55 of 115 [47.8%]). Patients with EGC and CH-PD exhibited a significantly worse median OS of 16.0 (95% CI, 11.6-22.3) months compared with 21.6 (95% CI, 19.6-24.3) months for those without CH-PD (*P* = .01). For patients with CRC and EGC, CH and CH-PD were not associated with PFS differences in patients undergoing ICB or first-line chemotherapy. Neither CH nor CH-PD were correlated with baseline leukocyte levels or increased need for G-CSF support.

**Conclusions and Relevance:**

These findings suggest CH and CH-PD are not directly associated with the treatment course of patients with metastatic gastrointestinal tract cancer receiving cancer-directed therapy.

## Key Points

**Question:**

To what extent is clonal hematopoiesis (CH) associated with treatment response and toxicity among patients with metastatic cancer who receive chemotherapy or immune checkpoint blockade?

**Findings:**

In this cohort study of 633 patients with metastatic esophagogastric and colorectal cancers, one-third of patients had CH, and half of these alterations were present in putative CH driver genes (CH-PD). The presence of CH and CH-PD were not associated with differences in progression-free survival, baseline leukocyte counts, or increased need for granulocyte colony-stimulating factor support.

**Meaning:**

Detection of CH or CH-PD does not appear to be associated with progression-free survival during chemotherapy or immune checkpoint blockade, nor with leukocyte recovery, suggesting limited utility of CH in solid tumor clinical decision-making.

## Teaser

This cohort study assesses the association of clonal hematopoiesis with progression-free and overall survival among patients with metastatic colorectal and esophagogastric cancers who were undergoing treatment with first-line chemotherapy or immunotherapy.

## Introduction

Clonal hematopoiesis (CH) represents nonrandom clonal selection of bone marrow–derived cells identified by somatic alterations in certain genes. A subset of these CH alterations occur in putative driver genes (CH-PD) known to be associated with hematologic neoplasia, often with a variant allele frequency of greater than 2%.^1^ The prevalence of CH increases with age^2^ and is affected by factors such as toxic exposures (eg, chemotherapy, radiotherapy, smoking).^3,4,5^ Presence of CH has been associated with the development of therapy-related myeloid neoplasms, increased risk of coronary heart disease and stroke, and inferior survival among patients with cancer.^3,6^ Preclinical evidence suggests that CH in hematopoietic stem cells (HSCs) induces a dysregulated inflammatory response as evidenced by altered inflammatory signaling,^7,8,9,10,11^ cytokine expression,^8,12,13,14^ and HSC differentiation.^15^

However, the clinical implications of these acquired leukocyte alterations in solid tumor therapy remain poorly understood, with conflicting trends in overall survival among patients with CH.^3,16^ In the context of the widespread use of immune checkpoint blockade (ICB) and the central role that HSC-derived lymphocytes play in mediating antitumor responses,^17,18^ we studied the association between CH and therapeutic efficacy and hematologic toxicity. We therefore retrospectively assessed the association of CH with progression-free survival (PFS) and overall survival (OS) in patients with metastatic colorectal (CRC) and esophagogastric (EGC) cancers who were undergoing treatment with first-line chemotherapy or immunotherapy.

## Methods

### Patients and Data

This retrospective cohort study included patients diagnosed with CRC or EGC between January 1, 2006, and December 31, 2020, at Memorial Sloan Kettering Cancer Center (MSKCC) who underwent sequencing of tumor and normal blood buffy coat samples using Memorial Sloan Kettering–Integrated Mutation Profiling of Actionable Cancer Targets (MSK-IMPACT),^19^ and who had available clinical and genomic data. All patients provided written informed consent to an institutional prospective tumor sequencing protocol,^20^ and this study was approved by the MSKCC institutional review board. This report followed the Strengthening the Reporting of Observational Studies in Epidemiology (STROBE) reporting guideline.

Clinical data including date of birth, smoking history, cancer history, age, date of death or last contact, date of diagnosis, date and duration of therapies, therapy type, date of progression, stage at diagnosis, and mismatch repair protein status (immunohistochemical expression for *PMS2*, *MLH1*, *MSH2*, and *MSH6*), were extracted from the institutional databases and clinical records by 3 abstractors (B.H.D., S.S., and S.B.M.) with random secondary cross-validation. Because of potential differences in CH based on race or ethnicity, patients’ self-reported race and ethnicity were collected from the electronic medical record. Variant calls were obtained from MSK-IMPACT testing, as were extrapolated tumor purity, alteration count, tumor alteration burden (TAB), and microsatellite instability (MSI) sensor score.^21^ We defined MSI-high (MSI-H) as having absence of mismatch repair immunohistochemical expression, with MSIsensor scores of at least 10^21,22^; for MSI-indeterminant samples (by MSIsensor), a multiple instance MSI score^23^ of at least 10 was used.

Baseline hematologic parameters were included for patients who had undergone a complete blood cell count with differential at MSKCC within 7 days prior to first-line chemotherapy initiation. Similarly, receipt of filgrastim or pegfilgrastim was retrieved from the electronic medical record for patients who received their first-line systemic therapy at MSKCC. The proportion of cycles of doublet (ie, FOLFOX [leucovorin (folinic acid), fluorouracil, and oxaliplatin], FOLFIRI [leucovorin (folinic acid), fluorouracil, and irinotecan]) or triplet (FLOT [fluorouracil, leucovorin (folinic acid), oxaliplatin, and docetaxel]) chemotherapy cycles requiring filgrastim or pegfilgrastim was calculated to account for variable chemotherapy intensity and duration, as well as differences in the number of doses of filgrastim vs longer-acting pegfilgrastim that would be utilized per cycle.

### CH Identification

Blood was collected prior to first-line chemotherapy initiation in 160 of 330 patients (48.5%) with EGC and 136 of 300 patients (45.3%) with CRC and prior to ICB initiation in 135 of 164 patients (82.3%) with EGC and 103 of 119 patients (86.6%) with CRC. The presence of CH was detected as previously described.^19,24^ Briefly, CH was defined per prior institutional publications as any alteration with a variant allele frequency of at least 2% and present in at least 10 reads, with a ratio of blood to tumor reads of at least 2:1 or a ratio of blood to lymph node of at least 1.5:1 that was not found in gnomAD (Genome Aggregation Database) with a frequency greater than 0.005. Variants were annotated as likely putative drivers (CH-PD) if they fulfilled any of multiple criteria, including oncogenic or likely oncogenic by OncoKB (Oncology Knowledge Base), truncating alterations in known tumor suppressor genes, reported as somatic at least 20 times in the Catalogue of Somatic Mutations in Cancer, or known myeloid and/or hematologic alterations in malignant neoplasms.^24^

### Study Outcomes

We calculated OS and PFS from the date of treatment start until the date of death (for OS) or until first treatment-related progression or death (for PFS), whichever occurred first. Patients who finished treatment without progression and continued to other definitive treatments (surgery, radiotherapy, or sought treatment elsewhere) were censored at their last date of evaluation, and the remaining patients who did not experience an event of interest for PFS were censored at their last contact date. For PFS analyses, first-line treatment was defined as first chemotherapy-containing treatment after stage IV cancer diagnosis. Patients who received ICB (without concurrent chemotherapy) as the first-line treatment were assessed for PFS only in the ICB cohort, not in the first-line chemotherapy cohort. For ICB PFS analyses, patients treated with ICB and concurrent chemotherapy were excluded from analysis to ensure homogeneous treatment populations (eTable 1 in Supplement 1).

### Statistical Analysis

All survival analyses were performed separately among the EGC and CRC cohorts based on the type of treatment received (first-line systemic therapy vs ICB). Baseline characteristics were compared between the presence of CH and CH-PD using the Wilcoxon rank sum test for continuous covariates and χ^2^ test for categorical variables. Fisher exact test was used for subgroups with numbers less than 5. We estimated OS and PFS using Kaplan-Meier methods and compared the presence of CH and CH-PD using a log-rank test. A Cox proportional hazards regression model was used to examine the association between CH-PD status and other baseline characteristics listed above with OS and PFS. Age at diagnosis and TAB were used as continuous variables in the models. Multivariable Cox proportional hazards regression was constructed for patients in the EGC–first-line systemic therapy cohort to evaluate the independent association between CH-PD and OS. Adjustment was made by including baseline clinical characteristics that were correlated with OS on univariable analysis at *P* < .20: age at diagnosis, prior treatment, *ERBB2* (previously known as *HER2*) status, and MSI status. Tumor alteration burden was not included in the multivariable model because it was highly correlated with MSI status. In the multivariable model, the hazard ratio (HR) was per 10-year increase in age. All *P* values were based on 2-tailed statistical analyses, and *P* ≤ .05 indicated statistical significance. All analyses were performed using R, version 4.0.4 (R Program for Statistical Computing).

## Results

**Figure 1.**
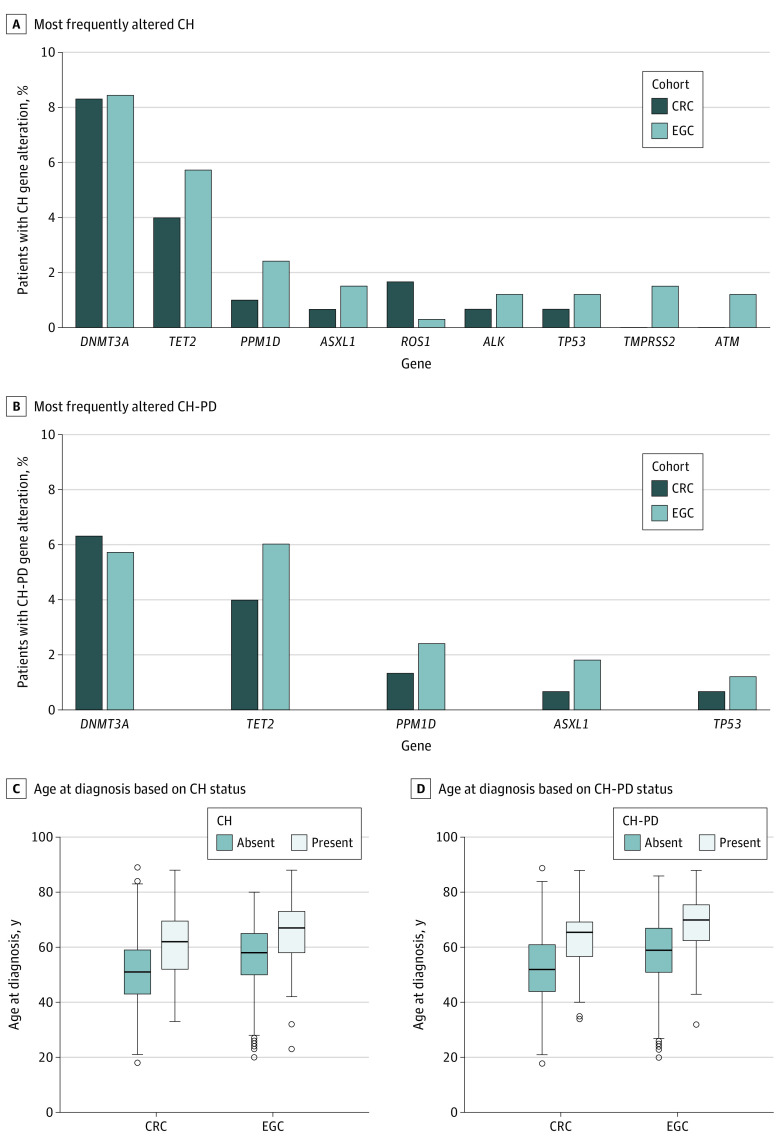
Characteristics of Clonal Hematopoiesis (CH) and CH Alterations Annotated as Likely Putative Drivers (CH-PD) in Patients With Metastatic Esophagogastric Cancer (EGC) and Colorectal Cancer (CRC) In the CRC group (n = 301), CH and CH-PD were present in 83 (27.6%) and 44 (14.6%) patients, respectively. In the EGC group (n = 332), CH and CH-PD were present in 115 (34.6%) and 55 (16.6%) patients, respectively. The most frequently altered CH (A) and CH-PD (B) genes in the metastatic EGC and CRC groups (frequency, ≥0.01) and age at diagnosis for EGC and CRC groups based on CH (C) and CH-PD (D) status are shown.

Of the 633 patients included in the analysis (390 men [61.6%] and 243 women (38.4%); median age, 58 [IQR, 48-66] years; 62 [9.8%] Asian, 45 [7.1%] Black or African American, 482 [76.1%] White, and 44 [7.0%] unknown race or ethnicity), we identified 332 patients with metastatic EGC (median age, 61 [IQR, 53-69] years; 229 men [69.0%] and 103 women [31.0%]) and 301 patients with metastatic CRC (median age, 52 [IQR, 45-63] years; 161 men [53.5%] and 140 women [46.5%]) (Table 1). Clinical and CH data were available for 630 patients (330 with EGC and 300 with CRC) who received first-line systemic therapy and 283 patients (164 with EGC and 119 with CRC) who received ICB therapy (Table 1 and eTable 1 in Supplement 1). Clonal hematopoiesis was present in 115 of 332 patients with EGC (34.6%) and 83 of 301 with CRC (27.6%); CH-PD was present in 55 of 332 with EGC (16.6%) and 44 of 301 with CRC (14.6%), such that approximately half of all patients with CH present had CH-PD (CRC group, 44 of 83 [53.0%]; EGC group, 55 of 115 [47.8%]). There was no significant difference in the proportion of the most frequently altered genes between EGC and CRC cohorts (*DNMT3A* [OMIM 602769], *TET2* [OMIM 612839], *PPM1D* [OMIM 605100], *ASXL1* [OMIM 612990], *ROS1* [OMIM 165020], *ALK* [OMIM 105590], *TP53* [OMIM 191170], *TMPRSS2* [OMIM 602060], and *ATM* [OMIM 607585]) (Figure 1A-B and eTable 2 in Supplement 1).

**Table 1.** Demographic and Clinical Characteristics of the EGC and CRC Groups

Characteristic	Patient group^a^	
	CRC (n = 301)	EGC (n = 332)
Age at diagnosis, median (IQR), y	52 (45-63)	61 (53-69)
Sex		
Men	161 (53.5)	229 (69.0)
Women	140 (46.5)	103 (31.0)
Stage at diagnosis		
I	3 (1.0)	3 (0.9)
II	9 (3.0)	17 (5.1)
III	33 (11.0)	62 (18.7)
IV	256 (85.0)	250 (75.3)
Race and ethnicity		
Asian	29 (9.6)	33 (9.9)
Black or African American	27 (9.0)	18 (5.4)
White	225 (74.8)	257 (77.4)
Unknown^b^	20 (6.6)	24 (7.2)
Primary site		
Right proximal	92 (30.6)	152 (45.8)
Left distal	203 (67.4)	179 (53.9)
Unknown	6 (2.0)	1 (0.3)
Prior chemotherapy^c^	188 (62.5)	217 (65.4)
Prior radiotherapy^c^	33 (11.0)	78 (23.5)
Treatment cohort^d^		
First-line systematic treatment	300 (99.7)	330 (99.4)
ICB	119 (39.5)	164 (49.4)
Current or former smoker	128 (42.5)	177 (53.3)
MMR status		
MSI-H	41 (13.6)	20 (6..0)
MSS	250 (83.1)	286 (80.7)
Unknown	10 (3.3)	26 (7.8)
*ERBB2* status		
Positive	NA	78 (23.5)
Negative	NA	250 (75.3)
Unknown	NA	4 (1.2)
PD-L1 status		
CPS≥1	NA	127 (38.3)
CPS<1	NA	125 (37.7)
Unknown	NA	80 (24.1)

Abbreviations: CPS, combined positive score; CRC, colorectal cancer; EGC, esophagogastric cancer; ICB, immune checkpoint blockade; MMR, mismatch repair; MSI-H, microsatellite instability–high; MSS, microsatellite stable; NA, not applicable; PD-L1, programmed cell death ligand 1.

^a^ Unless otherwise indicated, data are expressed as No. (%) of patients.

^b^ Indicates that race and ethnicity were not reported or the patient was of multiple races or ethnicities that could not be assigned to another category.

^c^ Relative to date of blood collection for next-generation sequencing and clonal hematopoiesis analysis.

^d^ Patients treated with ICB with first-line systemic treatment data available were analyzed in both cohorts. One patient in the CRC cohort and 2 in the EGC cohort receiving ICB did not have first-line treatment data available.

First, we evaluated the association between CH and CH-PD and patient demographic characteristics. Compared with patients without CH or CH-PD, the median age at diagnosis for patients with CH (CRC group, 62 [IQR, 52-70] vs 51 [IQR, 43-59] years; EGC group, 67 [IQR, 58-73] vs 58 [IQR, 50-65] years) and CH-PD (CRC group, 66 [IQR, 57-69] vs 52 [IQR, 44-61] years; EGC group, 70 [IQR, 62-76] vs 59 [IQR, 51-67] years) was significantly older (*P* < .001) in both cohorts (Figure 1C-D and eTable 3 in Supplement 1). Positive smoking history was associated with CH (74 of 115 [64.3%] vs 103 of 217 [47.5%]; *P* = .003) and CH-PD (39 of 55 [70.9%] vs 138 of 277 [49.8%]; *P* = .004) among patients with metastatic EGC. For patients with metastatic CRC, CH was more frequent in patients with MSI-H tumors (18 of 83 [21.7%] vs 23 of 218 [10.6%]; *P* = .01) and increased median TAB (7 [IQR, 5-11] vs 6 [IQR, 4-9]; *P* = .02) (eTable 3 in Supplement 1), though these patients tended to be older.

**Figure 2.**
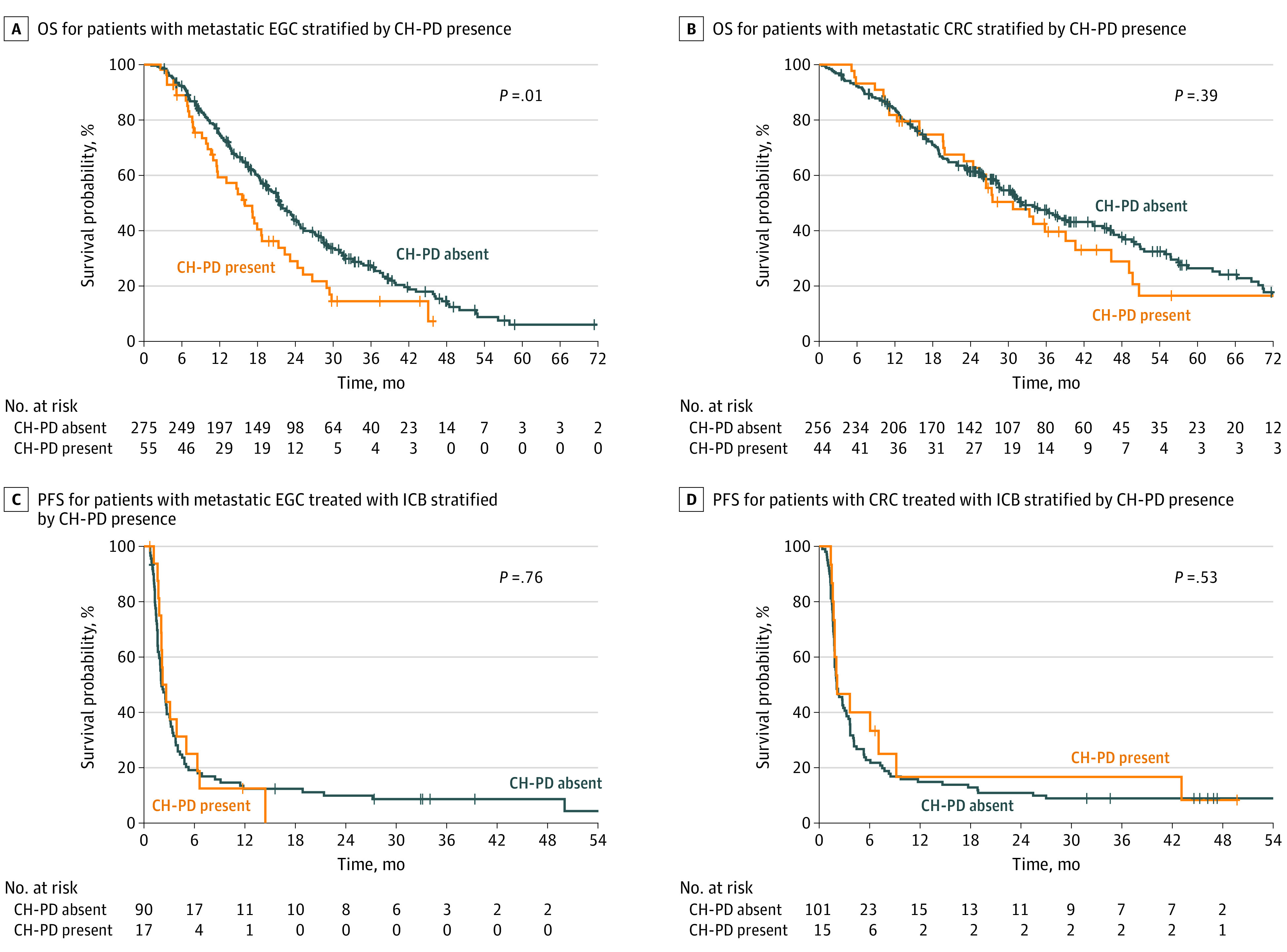
Survival Outcomes for Patients With Clonal Hematopoiesis (CH) Alterations Annotated as Likely Putative Drivers (CH-PD) Kaplan-Meier curves are shown for overall survival (OS) for patients with metastatic esophagogastric cancer (EGC) and colorectal cancer (CRC) based on CH-PD status (A and B) and for progression-free survival (PFS) among patients with metastatic EGC and CRC who were treated with immune checkpoint blockade (ICB), based on CH-PD status (C and D). Kaplan-Meier estimates of OS and PFS were compared using a log-rank test.

We then assessed whether CH or CH-PD was associated with differences in OS and PFS following first-line systemic therapy. Presence of CH was not significantly associated with OS for patients with EGC or CRC (eFigure 1A-B in Supplement 1). However, when stratifying patients by CH-PD status, those with EGC and CH-PD exhibited a significantly worse median OS (16.0 [95% CI, 11.6-22.3] months) compared with those without CH-PD (21.6 [95% CI, 19.6-24.3] months; *P* = .01) (Figure 2A). Multivariable analysis showed that CH-PD presence was independently associated with worse OS (HR, 1.52 [95% CI, 1.06-2.20]; *P* = .02) after accounting for age, prior therapies (radiotherapy and/or chemotherapy) and tumor characteristics (*ERBB2* status and MSI-H) (Table 2). Surprisingly, prior chemotherapy (HR, 0.69 [95% CI, 0.52-0.93]; *P* = .01) was also independently associated with improved OS for patients with EGC. Among patients with metastatic CRC, no association was found between CH-PD and OS on univariable analysis (HR, 1.18 [95% CI, 0.81-1.72]; *P* = .39) (Figure 2B). However, again, prior radiotherapy and chemotherapy (HR, 0.38 [95% CI, 0.23-0.63]; *P* < .001) or chemotherapy alone (HR, 0.53 [95% CI, 0.39-0.72]; *P* < .001) were associated with improved OS (Table 3). We also assessed OS from the date of blood collection or first-line systemic treatment initiation, whichever was later, in lieu of calculating from first-line systemic treatment initiation as above. Comparable results were achieved, with those with EGC and CH-PD exhibiting significantly worse median OS (19.2 [95% CI, 17.5-21.6] vs 13.1 [95% CI, 9.2-18.7] months for CH-PD absent vs present; *P* = .02) (eFigure 2A-B in Supplement 1). For patients in the CRC and EGC cohorts undergoing first-line systemic therapy, there were no differences in PFS based on CH or CH-PD status (eFigures 1C-D and 3A-D in Supplement 1).

**Table 2.** Univariable and Multivariable Analysis of CH-PD and OS Among Patients With Metastatic EGC Using a Cox Proportional Hazards Regression Model

Characteristic	Univariable OS analysis			Multivariable OS analysis	
	No. of patients	HR (95% CI)	*P* value	HR (95% CI)	*P* value
CH-PD					
Absent	330	1 [Reference]	NA	NA	NA
Present		1.54 (1.11-2.16)	.02	1.52 (1.06-2.20)	.02
Diagnosis age	330	1.01 (1.00-1.02)	.09	1.10 (0.99-1.23)	.08
Smoking history					
Never smoker	330	1 [Reference]	NA	NA	NA
Current/former		1.17 (0.91-1.51)	.23		
Prior RT or chemotherapy			.12		
None	330	1 [Reference]	NA	NA	NA
Prior RT and chemotherapy		0.75 (0.52-1.07)		0.75 (0.51-1.09)	.13
Prior chemotherapy only		0.69 (0.52-0.93)		0.68 (0.50-0.93)	.01
Prior RT only		0.79 (0.29-2.16)		0.96 (0.35-2.64)	>.90
*ERBB2* status					
Negative	330	1 [Reference]	NA	NA	NA
Positive		0.64 (0.47-0.87)	.003	0.58 (0.42-0.80)	<.001
MMR status					
MSS	303	1 [Reference]	NA	NA	NA
MSI-H		0.49 (0.27-0.90)	.01	0.38 (0.21-0.72)	.003
TAB	328	0.985 (0.971-0.998)	.01	NA	NA

Abbreviations: CH-PD, clonal hematopoiesis alterations annotated as likely putative drivers; HR, hazard ratio; MMR, mismatch repair; MSI-H, microsatellite instability–high; MSS, microsatellite stable; NA, not applicable; OS, overall survival; RT, radiotherapy; TAB, tumor alteration burden.

**Table 3.** Univariable Analysis of CH-PD and OS Among Patients With Metastatic CRC

Characteristic	Univariable OS analysis		
	No. of patients	HR (95% CI)	*P* value
CH-PD			
Absent	300	1 [Reference]	NA
Present		1.18 (0.81-1.72)	.39
Diagnosis age	300	1.01 (1.00-1.02)	.11
Smoking history			
Never smoker	300	1 [Reference]	NA
Current/former		1.04 (0.78-1.38)	.78
Prior RT or chemotherapy			
None	300	1 [Reference]	NA
Prior			
RT and chemotherapy		0.38 (0.23-0.63)	<.001
Prior chemotherapy only		0.53 (0.39-0.72)	<.001
RT only		2.69 (0.37-19.7)	.33
MMR status			
MSS	290	1 [Reference]	NA
MSI-H		0.66 (0.42-1.05)	.08
TAB	300	0.995 (0.988-1.001)	.11

Abbreviations: CH-PD, clonal hematopoiesis alterations annotated as likely putative drivers; CRC, colorectal cancer; MMR, mismatch repair; MSI-H, microsatellite instability–high; MSS, microsatellite stable; NA, not applicable; OS, overall survival; RT, radiotherapy; TAB, tumor alteration burden.

We then evaluated the immune-modulating potential of CH by assessing the association between CH and ICB outcomes. Upon univariable analysis, there was no significant difference in PFS when stratified by presence of CH (eFigure 1E-F in Supplement 1) or CH-PD (Figure 2C-D) among patients in both the EGC and CRC cohorts receiving ICB therapy, while MSI-H status was associated with improved PFS for the EGC (HR, 0.30 [95% CI, 0.14-0.62]; *P* = .001) and CRC (HR, 0.22 [95% CI, 0.13-0.38]; *P* < .001) cohorts, as would be expected (eFigure 4A-B in Supplement 1).

Many patients with EGC and CRC require dose reductions in their doublet and triplet chemotherapy and/or granulocyte colony-stimulating factor (G-CSF) support to continue therapy. Therefore, we next sought to evaluate the association of CH and CH-PD presence with baseline leukocyte count and composition prior to initiating first-line doublet or triplet chemotherapy. There was no significant difference in white blood cell count, absolute neutrophil count, absolute lymphocyte count, or neutrophil-to-lymphocyte ratio between patients with or without CH or CH-PD before initiating first-line systemic therapy, suggesting that CH did not impair hematopoiesis (eFigure 5A-H in Supplement 1).

We then assessed whether the presence of CH and CH-PD was associated with the need for G-CSF support during first-line systemic therapy, specifically the proportion of platinum- or irinotecan-containing cycles that required G-CSF support. Presence of CH was not associated with significant differences in the proportion of cycles requiring G-CSF support in either cohort. Among patients with CRC, those with CH-PD required fewer chemotherapy cycles with G-CSF support (median proportion of first-line chemotherapy cycles requiring G-CSF: 0.11 [IQR, 0-0.33] vs 0 [IQR, 0-0.06]; *P* = .007), while for patients with EGC, there was no difference in G-CSF support based on CH-PD presence (eFigure 6A-B in Supplement 1). However, we noted that in the CRC cohort, no patients with CH-PD received triplet chemotherapy, likely related to their older age at diagnosis, compared with patients without CH-PD, of whom 15 (7.3%) received triplet chemotherapy, which may explain why patients without CH-PD appeared to require more G-CSF support.

## Discussion

In this cohort study, while CH-PD was associated with reduced OS among patients with metastatic EGC, CH and CH-PD were not associated with PFS in patients receiving first-line systemic therapy or ICB. Previous pancancer studies^3^ suggested inferior OS for patients with CH-PD, but these analyses failed to account for phenotypes with outlying therapeutic benefit (*ERBB2*, MSI-H, etc) or cancer stage at diagnosis. By including patients with earlier-stage cancer, their results may drastically overestimate survival in the recurrent or de novo stage IV setting. In contrast, a CRC-specific evaluation of patients treated with first-line FOLFIRI plus cetuximab or bevacizumab in the FIRE-3 trial^16^ suggested that patients with CH, in particular *DNMT3A* alterations, actually had an improved OS but no differences in PFS in this treatment setting. Our study corroborates these findings with lack of difference based on CH status across multiple first-line systemic therapies in EGC and CRC, while also demonstrating this with ICB. This finding may reflect inadequate exposure or lead time from CH-accelerating agents (smoking, radiotherapy, chemotherapy) to CH development in patients with limited life expectancy. However, our findings, particularly in EGC, suggest that CH may primarily reflect older patients with more comorbidities, and thus inferior prognosis due to competing risks, rather than CH-related morbidity. Additionally, we demonstrated that CH and CH-PD were not associated with pretreatment leukopenia or increased need for G-CSF support. These findings suggest that the mere presence of CH or CH-PD does not significantly impair leukocyte proliferation or function, in either lymphoid or myeloid lineages, in a clinically meaningful manner.

### Limitations

Our study has several limitations. The EGC and CRC cohorts represent heterogeneously treated patients with samples collected for CH analysis at variable milestones during their treatment course. Therefore, receipt of prior chemotherapy and/or radiotherapy may increase the incidence of CH and CH-PD in these patients with delayed blood collection. To address this, we also performed analyses assessing OS from the date of blood collection (for next-generation sequencing and CH analysis) or first-line systemic treatment initiation, whichever was later, instead of calculating from first-line systemic treatment initiation, which showed similar results (eFigure 2A-B in Supplement 1). In addition, some associations we identified, such as MSI-H being associated with CH presence, may reflect higher incidences of both sporadic MSI and CH among older patients with cancer.^25,26^ Additional CH-associated conditions such as hematologic neoplasms, cardiac events, and neurological events were not evaluated, as most patients had limited follow-up time due to competing risk from cancer-related mortality.^3,6^

## Conclusions

Our findings suggest that CH is not associated with differences in PFS for patients receiving cancer-directed therapy, with leukocyte quantity, or with increased need for G-CSF support. Together these findings suggest limited utility of CH in solid tumor clinical decision-making.

## Notes

Supplement 1. **eTable 1.** ICB Types Utilized for Treatment **eTable 2.** Most Commonly Altered CH and CH-PD Genes **eTable 3.** Association Between Patient and Tumor Features With CH and CH-PD **eFigure 1.** Survival Trends for Patients With EGC and CRC Based on CH Status **eFigure 2.** Overall Survival Trend for Patients With EGC and CRC Based on CH-PD Status From Date of Blood Draw (DOP) **eFigure 3.** PFS Trend for Patients With EGC and CRC Receiving First-Line Treatment Based on CH-PD Status **eFigure 4.** Univariable Analyses for PFS **eFigure 5.** No Difference in WBC and Subtype Cell Populations for Metastatic GI Cancer Cohorts Based on CH and CH-PD Status **eFigure 6.** G-CSF Support Based on CH or CH-PD Status

Supplement 2.
Data Sharing Statement

## References

[1] Steensma DP, Bejar R, Jaiswal S, . Clonal hematopoiesis of indeterminate potential and its distinction from myelodysplastic syndromes. Blood. 2015;126(1):9-16. doi:10.1182/blood-2015-03-631747 25931582 PMC4624443

[2] Genovese G, Kähler AK, Handsaker RE, . Clonal hematopoiesis and blood-cancer risk inferred from blood DNA sequence. N Engl J Med. 2014;371(26):2477-2487. doi:10.1056/NEJMoa1409405 25426838 PMC4290021

[3] Coombs CC, Zehir A, Devlin SM, . Therapy-related clonal hematopoiesis in patients with non-hematologic cancers is common and associated with adverse clinical outcomes. Cell Stem Cell. 2017;21(3):374-382.e4. doi:10.1016/j.stem.2017.07.010 28803919 PMC5591073

[4] Hsu JI, Dayaram T, Tovy A, . *PPM1D* mutations drive clonal hematopoiesis in response to cytotoxic chemotherapy. Cell Stem Cell. 2018;23(5):700-713.e6. doi:10.1016/j.stem.2018.10.004 30388424 PMC6224657

[5] Wong TN, Ramsingh G, Young AL, . Role of *TP53* mutations in the origin and evolution of therapy-related acute myeloid leukaemia. Nature. 2015;518(7540):552-555. doi:10.1038/nature13968 25487151 PMC4403236

[6] Jaiswal S, Fontanillas P, Flannick J, . Age-related clonal hematopoiesis associated with adverse outcomes. N Engl J Med. 2014;371(26):2488-2498. doi:10.1056/NEJMoa1408617 25426837 PMC4306669

[7] Cai Z, Kotzin JJ, Ramdas B, . Inhibition of inflammatory signaling in *Tet2* mutant preleukemic cells mitigates stress-induced abnormalities and clonal hematopoiesis. Cell Stem Cell. 2018;23(6):833-849.e5. doi:10.1016/j.stem.2018.10.013 30526882 PMC6317370

[8] Fuster JJ, MacLauchlan S, Zuriaga MA, . Clonal hematopoiesis associated with *TET2* deficiency accelerates atherosclerosis development in mice. Science. 2017;355(6327):842-847. doi:10.1126/science.aag1381 28104796 PMC5542057

[9] Hormaechea-Agulla D, Matatall KA, Le DT, . Chronic infection drives Dnmt3a-loss-of-function clonal hematopoiesis via IFNγ signaling. Cell Stem Cell. 2021;28(8):1428-1442.e6. doi:10.1016/j.stem.2021.03.002 33743191 PMC8349829

[10] Meisel M, Hinterleitner R, Pacis A, . Microbial signals drive pre-leukaemic myeloproliferation in a *Tet2*-deficient host. Nature. 2018;557(7706):580-584. doi:10.1038/s41586-018-0125-z 29769727 PMC6238954

[11] Zekavat SM, Lin S-H, Bick AG, ; Biobank Japan Project; FinnGen Consortium. Hematopoietic mosaic chromosomal alterations increase the risk for diverse types of infection. Nat Med. 2021;27(6):1012-1024. doi:10.1038/s41591-021-01371-0 34099924 PMC8245201

[12] Cull AH, Snetsinger B, Buckstein R, Wells RA, Rauh MJ. *Tet2* restrains inflammatory gene expression in macrophages. Exp Hematol. 2017;55:56-70.e13. doi:10.1016/j.exphem.2017.08.001 28826859

[13] Sano S, Oshima K, Wang Y, . Tet2-mediated clonal hematopoiesis accelerates heart failure through a mechanism involving the IL-1β/NLRP3 inflammasome. J Am Coll Cardiol. 2018;71(8):875-886. doi:10.1016/j.jacc.2017.12.037 29471939 PMC5828038

[14] Zhang Q, Zhao K, Shen Q, . Tet2 is required to resolve inflammation by recruiting Hdac2 to specifically repress IL-6. Nature. 2015;525(7569):389-393. doi:10.1038/nature15252 26287468 PMC4697747

[15] Izzo F, Lee SC, Poran A, . DNA methylation disruption reshapes the hematopoietic differentiation landscape. Nat Genet. 2020;52(4):378-387. doi:10.1038/s41588-020-0595-4 32203468 PMC7216752

[16] Arends CM, Dimitriou S, Stahler A, . Clonal hematopoiesis is associated with improved survival in patients with metastatic colorectal cancer from the FIRE-3 trial. Blood. 2022;139(10):1593-1597. doi:10.1182/blood.2021014108 34932794

[17] McGranahan N, Furness AJS, Rosenthal R, . Clonal neoantigens elicit T cell immunoreactivity and sensitivity to immune checkpoint blockade. Science. 2016;351(6280):1463-1469. doi:10.1126/science.aaf1490 26940869 PMC4984254

[18] Ribas A, Wolchok JD. Cancer immunotherapy using checkpoint blockade. Science. 2018;359(6382):1350-1355. doi:10.1126/science.aar4060 29567705 PMC7391259

[19] Cheng DT, Mitchell TN, Zehir A, . Memorial Sloan Kettering–Integrated Mutation Profiling of Actionable Cancer Targets (MSK-IMPACT): a hybridization capture-based next-generation sequencing clinical assay for solid tumor molecular oncology. J Mol Diagn. 2015;17(3):251-264. doi:10.1016/j.jmoldx.2014.12.006 25801821 PMC5808190

[20] Genomic profiling in cancer patients. ClinicalTrials.gov identifier: NCT01775072. Updated December 29, 2022. Accessed January 12, 2023. https://clinicaltrials.gov/ct2/show/NCT01775072

[21] Niu B, Ye K, Zhang Q, . MSIsensor: microsatellite instability detection using paired tumor-normal sequence data. Bioinformatics. 2014;30(7):1015-1016. doi:10.1093/bioinformatics/btt755 24371154 PMC3967115

[22] Middha S, Zhang L, Nafa K, . Reliable pan-cancer microsatellite instability assessment by using targeted next-generation sequencing data. JCO Precis Oncol. 2017;2017:PO.17.00084. doi:10.1200/PO.17.00084 30211344 PMC6130812

[23] Ziegler J, Hechtman JF, Ptashkin R, . MiMSI—a deep multiple instance learning framework improves microsatellite instability detection from tumor next-generation sequencing. bioRxiv. Preprint posted online September 18, 2020. doi:10.1101/2020.09.16.299925 PMC11696176 39746944

[24] Bolton KL, Ptashkin RN, Gao T, . Cancer therapy shapes the fitness landscape of clonal hematopoiesis. Nat Genet. 2020;52(11):1219-1226. doi:10.1038/s41588-020-00710-0 33106634 PMC7891089

[25] Poynter JN, Siegmund KD, Weisenberger DJ, ; Colon Cancer Family Registry Investigators. Molecular characterization of MSI-H colorectal cancer by *MLHI* promoter methylation, immunohistochemistry, and mismatch repair germline mutation screening. Cancer Epidemiol Biomarkers Prev. 2008;17(11):3208-3215. doi:10.1158/1055-9965.EPI-08-0512 18990764 PMC2628332

[26] Velho S, Fernandes MS, Leite M, Figueiredo C, Seruca R. Causes and consequences of microsatellite instability in gastric carcinogenesis. World J Gastroenterol. 2014;20(44):16433-16442. doi:10.3748/wjg.v20.i44.16433 25469011 PMC4248186

