# Supplementary material for: Clinical Importance of Clonal Hematopoiesis in Metastatic Gastrointestinal Tract Cancers
Source: JAMA Netw Open. 2023 Feb 2;6(2):e2254221. doi: 10.1001/jamanetworkopen.2022.54221 (PMC9896303; doi:10.1001/jamanetworkopen.2022.54221)
Supplement: Supplement 1. — eTable 1. ICB Types Utilized for Treatment eTable 2. Most Commonly Altered CH and CH-PD Genes eTable 3. Association Between Patient and Tumor Features With CH and CH-PD eFigure 1. Survival Trends for Patients With EGC and CRC Based on CH Status eFigure 2. Overall Survival Trend for Patients With EGC and CRC Based on CH-PD Status From Date of Blood Draw (DOP) eFigure 3. PFS Trend for Patients With EGC and CRC Receiving First-Line Treatment Based on CH-PD Status eFigure 4. Univariable Analyses for PFS eFigure 5. No Difference in WBC and Subtype Cell Populations for Metastatic GI Cancer Cohorts Based on CH and CH-PD Status eFigure 6. G-CSF Support Based on CH or CH-PD Status [file jamanetwopen-e2254221-s001.pdf]

Supplementary Online Content

Diplas BH, Ptashkin R, Chou JF, et al. Clinical importance of clonal hematopoiesis in metastatic gastrointestinal tract cancers. *JAMA Netw Open*. 2023;6(2):e2254221. doi:10.1001/jamanetworkopen.2022.54221

eTable 1. ICB Types Utilized for Treatment
eTable 2. Most Commonly Altered CH and CH-PD Genes
eTable 3. Association Between Patient and Tumor Features With CH and CH-PD
eFigure 1. Survival Trends for Patients With EGC and CRC Based on CH Status
eFigure 2. Overall Survival Trend for Patients With EGC and CRC Based on CH-PD Status From Date of Blood Draw (DOP)
eFigure 3. PFS Trend for Patients With EGC and CRC Receiving First-Line Treatment Based on CH-PD Status
eFigure 4. Univariable Analyses for PFS
eFigure 5. No Difference in WBC and Subtype Cell Populations for Metastatic GI Cancer Cohorts Based on CH and CH-PD Status
eFigure 6. G-CSF Support Based on CH or CH-PD Status

This supplementary material has been provided by the authors to give readers additional information about their work.

© 2023 Diplas BH et al. *JAMA Network Open*.

SUPPLEMENTARY TABLES

eTable 1. ICB Types Utilized for Treatment

**Regimen**	**Colorectal cancer (CRC)** n=119 No. (%)	**Esophagogastric cancer (EGC)** n=164 No. (%)
PD-1i	66 (55%)	64 (39%)
CTLA-4i	0 (0%)	1 (0.6%)
PD-1i + CTLA-4i	12 (10%)	34 (21%)
PD-1i + investigational therapy	22 (18%)	6 (3.7%)
PD-1i + targeted therapy	13 (11%)	2 (1.2%)
PD-1i + targeted + investigational therapy	3 (2.5%)	0 (0%)
PD-1i + chemotherapy†	3 (2.5%)	13 (7.9%)
PD-1i + chemotherapy + targeted therapy†	0 (0%)	44 (27%)
†*Not included in PFS analysis*		
PD-1i could represent PD-1 or PD-L1 targeting therapy		

© 2023 Diplas BH et al. *JAMA Network Open*.

eTable 2. Most Commonly Altered CH and CH-PD Genes

CH	CRC		EGC	
	N	%	N	%
DNMT3A	25	8.31	28	8.43
TET2	12	3.99	19	5.72
PPM1D	3	1.00	8	2.41
ASXL1	2	0.66	5	1.51
ROS1	0	0.00	5	1.51
ALK	2	0.66	4	1.20
TP53	0	0.00	4	1.20
TMPRSS2	2	0.66	4	1.20
ATM	5	1.66	1	0.30
**CH-PD**	**CRC**		**EGC**	
	**N**	**%**	**N**	**%**
DNMT3A	12	3.99	20	6.02
TET2	19	6.31	19	5.72
PPM1D	4	1.33	8	2.41
ASXL1	2	0.66	6	1.81
TP53	2	0.66	4	1.20

© 2023 Diplas BH et al. *JAMA Network Open*.

eTable 3. Association Between Patient and Tumor Features With CH and CH-PD

**Colorectal cancer**<br>n = 301<br>**Characteristic**	**No CH**<br>n = 218<br>No. (%)	**CH**<br>n = 83<br>No. (%)	**p-value**[1]	**No CH-PD**<br>n = 257<br>No. (%)	**CH-PD**<br>n = 44<br>No. (%)	**p-value**[1]
Median age at diagnosis, years (IQR)	51 (43, 59)	62 (52, 70)	<0.001	52 (44, 61)	66 (57, 69)	<0.001
Smoking history			0.3			0.8
Never smoker	129 (59%)	44 (53%)		147 (57%)	26 (59%)	
Current/former smoker	89 (41%)	39 (47%)		110 (43%)	18 (41%)	
Prior radiation or chemotherapy			0.2			0.6
No prior chemotherapy or RT	83 (38%)	28 (34%)		91 (35%)	20 (45%)	
Prior chemotherapy and RT	22 (10%)	9 (11%)		27 (11%)	4 (9.1%)	
Prior chemotherapy only	113 (52%)	44 (53%)		137 (53%)	20 (45%)	
Unknown	0	2 (2.4%)		2 (0.8%)	0	
MMR status			0.011			0.12
MSS	188 (89%)	62 (78%)		218 (87%)	32 (78%)	
MSI-H	23 (11%)	18 (22%)		32 (13%)	9 (22%)	
Unknown	7	3		7	3	
Median TMB[1]	6 (4, 9)	7 (5, 11)	0.015	6 (4, 10)	7 (4, 12)	0.4
**Esophagogastric cancer**<br>n = 332<br>**Characteristic**	**No CH**<br>n = 217<br>No. (%)	**CH**<br>n = 115<br>No. (%)	**p-value**[1]	**No CH-PD**<br>n = 277<br>No. (%)	**CH-PD**<br>n = 55<br>No. (%)	**p-value**[1]
Median age at diagnosis, years (IQR)	58 (50, 65)	67 (58, 73)	<0.001	59 (51, 67)	70 (62, 76)	<0.001
Smoking history			0.003			0.004
Never smoker	114 (53%)	41 (36%)		139 (50%)	16 (29%)	
Current/former smoker	103 (47%)	74 (64%)		138 (50%)	39 (71%)	
Prior radiation or chemotherapy			0.2			0.5
No prior chemotherapy or RT	70 (32%)	37 (32%)		86 (31%)	21 (38%)	
Prior chemotherapy and RT	39 (18%)	31 (27%)		57 (21%)	13 (24%)	
Prior chemotherapy only	103 (47%)	44 (38%)		126 (45%)	21 (38%)	
Prior RT only	5 (2.3%)	3 (2.6%)		8 (2.9%)		
HER2			0.3			0.2
HER2 negative	160 (74%)	90 (80%)		204 (75%)	46 (84%)	
HER2 positive	55 (26%)	23 (20%)		69 (25%)	9 (16%)	
Unknown	2	2		4	0	
MMR status			0.065			0.8
MSS	186 (95%)	98 (90%)		237 (94%)	47 (92%)	
MSI-H	9 (4.6%)	11 (10%)		16 (6.3%)	4 (7.8%)	
Unknown	22	6		24	4	
Median TMB (IQR)	4 (3, 7)	4 (3, 6)	0.2	4 (3, 7)	4 (3, 6)	0.7
Unknown	2	0		2	0	

Data are presented as No. (%) unless otherwise stated
[1]Wilcoxon rank sum test used for continuous variables; Pearson's Chi-squared test or Fisher's exact test used for binary variables

© 2023 Diplas BH et al. *JAMA Network Open*.

RT: Radiation treatment; MMR: Mismatch repair; MSS: Microsatellite stable; MSI-H: Microsatellite instability high; TMB: Tumor mutation burden

© 2023 Diplas BH et al. *JAMA Network Open*.

SUPPLEMENTARY FIGURE LEGENDS

eFigure 1. Survival Trends for Patients With EGC and CRC Based on CH Status. a,b, Kaplan-Meier curves of OS for EGC **(a)** and CRC **(b)** patients. **c,d,** Kaplan-Meier curves of PFS for EGC **(c)** and CRC **(d)** patients treated with 1L therapy. **e,f,** Kaplan-Meier curves of PFS for EGC **(e)** and CRC **(f)** patients treated with ICB. (Kaplan-Meier estimates of OS and PFS, log-rank test).

eFigure 2. Overall Survival Trend for Patients With EGC and CRC Based on CH-PD Status From Date of Blood Draw (DOP). a,b, Kaplan-Meier curves for OS for patients with metastatic EGC **(a)** and CRC **(b)**, with survival interval starting at date of blood draw (DOP) for CH-PD analysis or 1L treatment start date, whichever is later. (Kaplan-Meier estimates of OS, log-rank test).

eFigure 3. PFS Trend for Patients With EGC and CRC Receiving First-Line Treatment Based on CH-PD Status. a,b, Kaplan-Meier curves of PFS for patients with metastatic EGC **(a)** with univariable analysis for PFS **(b). c,d,** Kaplan-Meier curves of PFS for patients with metastatic CRC **(c)** with univariable analysis for PFS **(d).** TMB and age are continuous variables, with HR as per 1 unit increase in TMB and per 10-year increase in age. (Kaplan-Meier estimates of PFS, log-rank test).

eFigure 4. Univariable Analyses for PFS. a,b Univariable analysis for PFS for metastatic EGC **(a)** and CRC **(b)** patients treated ICB. TMB and age are continuous variables, with HR as per 1 unit increase in TMB and per 10-year increase in age.

eFigure 5. No Difference in WBC and Subtype Cell Populations for Metastatic GI Cancer Cohorts Based on CH and CH-PD Status. WBC and subtype cell counts data obtained prior to 1st line therapy and analyzed for differences based on CH and CH-PD status. Data was available for 68.3% (205/300) of CRC and 76.1% (251/330) of EGC patients. **a,b,** White blood cell (WBC) for EGC **(a)** and CRC **(b)** patients. **c,d**, Absolute neutrophil count (ANC), for EGC **(c)** and CRC **(d)** patients. e,f, Absolute lymphocyte count (ALC), for EGC **(e)** and CRC **(f)** patients. **g,h,** Neutrophil-to-lymphocyte ratio (NLR) for EGC **(g)** and CRC **(h)** patients. (Wilcoxon rank sum test). P values all greater than 0.05.

© 2023 Diplas BH et al. *JAMA Network Open*.

eFigure 6. G-CSF Support Based on CH or CH-PD Status. G-CSF support dose data was available for 80.3% (241/300) of CRC and 81.5% (269/330) of EGC patients during 1L therapy. **a,b,** Proportion of 1L chemotherapy cycles requiring G-CSF support for metastatic EGC and CRC patients based on CH **(a)** and CH-PD **(b)** status. None of the patients with CH-PD in the CRC cohort received triplet therapy whereas 7.3% of CH-PD negative patients received triplet therapy. (Wilcoxon rank sum test).

© 2023 Diplas BH et al. *JAMA Network Open*.

SUPPLEMENTARY FIGURES

eFigure 1. Survival Trends for Patients With EGC and CRC Based on CH Status

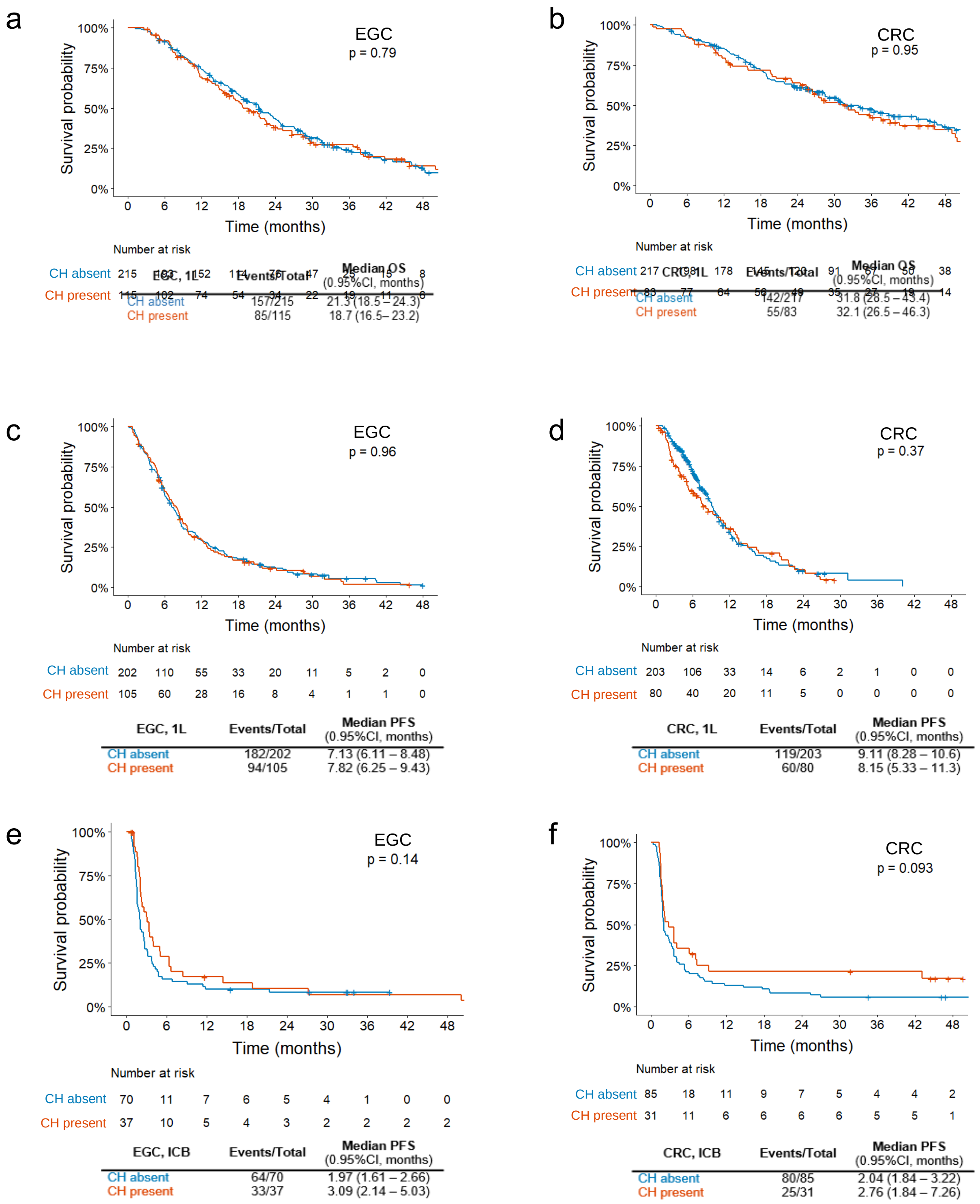

© 2023 Diplas BH et al. *JAMA Network Open*.

eFigure 2. Overall Survival Trend for Patients With EGC and CRC Based on CH-PD Status From Date of Blood Draw (DOP)

a

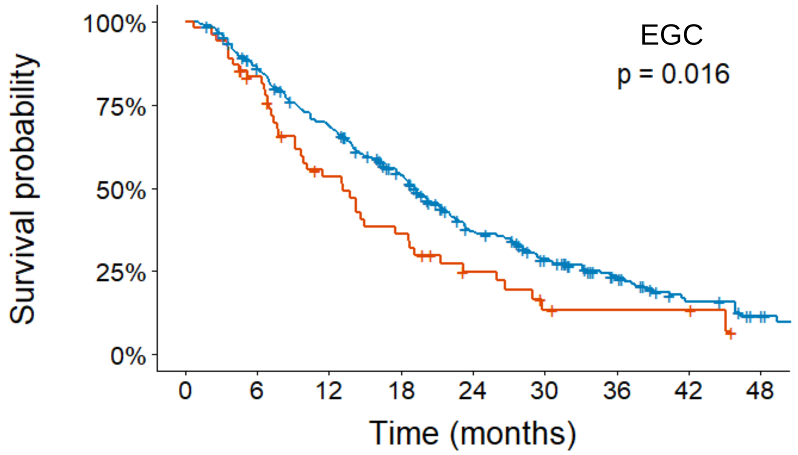

Number at risk

	0	6	12	18	24	30	36	42	48
CH-PD Absent	275	230	181	130	78	52	31	16	8
CH-PD Present	55	43	25	17	9	4	3	3	0

EGC, 1L	Events/Total	Median OS (0.95%CI, months)
CH-PD Absent	200/275	19.2 (17.5 – 21.6)
CH-PD Present	42/55	13.1 (9.2 – 18.7)

b

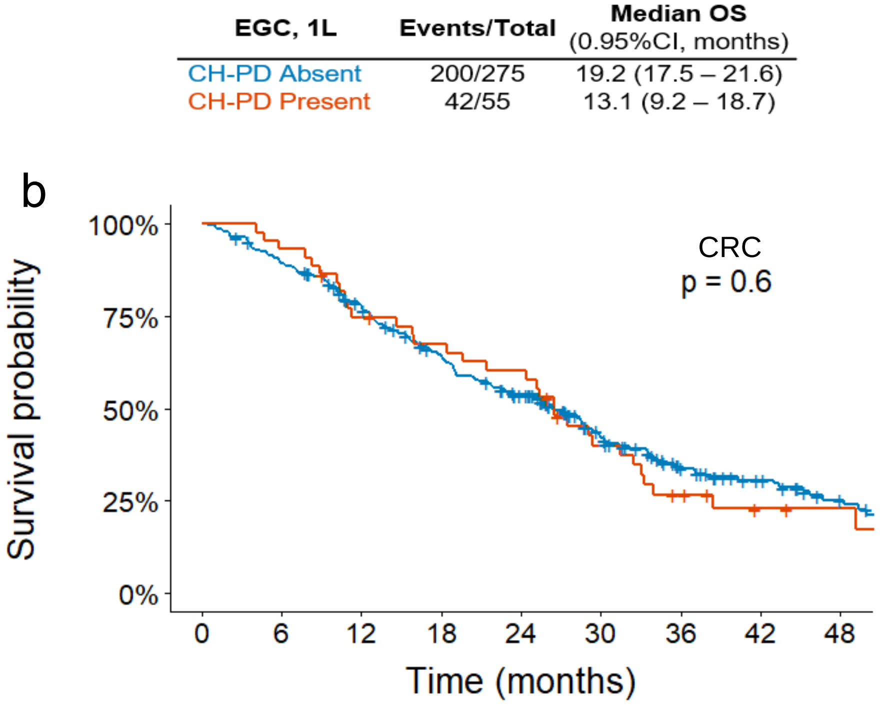

Number at risk

	0	6	12	18	24	30	36	42	48
CH-PD Absent	256	227	187	148	118	73	46	32	20
CH-PD Present	44	41	32	28	25	15	9	5	4

CRC, 1L	Events/Total	Median OS (0.95%CI, months)
CH-PD Absent	164/256	26.3 (21.9 – 29.8)
CH-PD Present	33/44	26.5 (19.6 – 33.2)

© 2023 Diplas BH et al. *JAMA Network Open*.

eFigure 3. PFS Trend for Patients With EGC and CRC Receiving First-Line Treatment Based on CH-PD Status

a

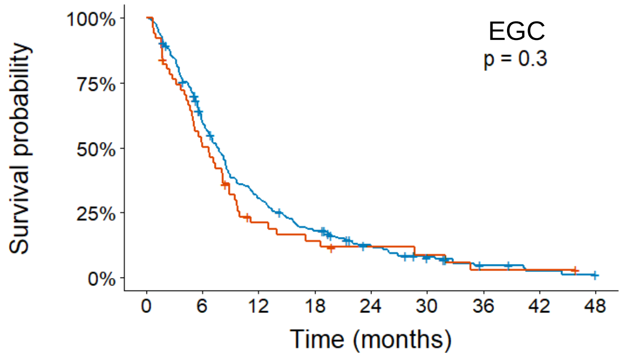

Number at risk

	0	6	12	18	24	30	36	42	48
CH-PD absent	256	145	74	43	24	12	5	2	0
CH-PD present	51	25	9	6	4	3	1	1	0

EGC, 1L	Events/Total	Median PFS (0.95%CI, months)
CH-PD absent	230/256	7.76 (6.64 – 8.51)
CH-PD present	46/51	6.71 (5.00 – 8.84)

b

Esophagogastric cancer, 1L

	**N**	**Univariate PFS** **HR (95% CI)**	**p-value**
CH-PD	307		
Absent		ref	—
Present		1.18 (0.86, 1.62)	0.3
Diagnosis Age	307	0.995 (0.985, 1.004)	0.24
Smoking History	307		
Never Smoker		ref	—
Current/Former		0.99 (0.78, 1.26)	0.95
Prior RT or Chemotherapy	307		
None		ref	—
Prior RT and Chemotherapy		0.94 (0.66, 1.33)	0.71
Prior Chemotherapy only		0.86 (0.65, 1.13)	0.27
Prior RT only		0.79 (0.35, 1.81)	0.58
HER2	307		
Negative		ref	—
Positive		0.53 (0.40, 0.71)	<0.001
MMR status	282		
MSS		ref	
MSI-H		1.98 (1.08, 3.63)	0.028
TMB	305	1.01 (0.99, 1.03)	0.23

c

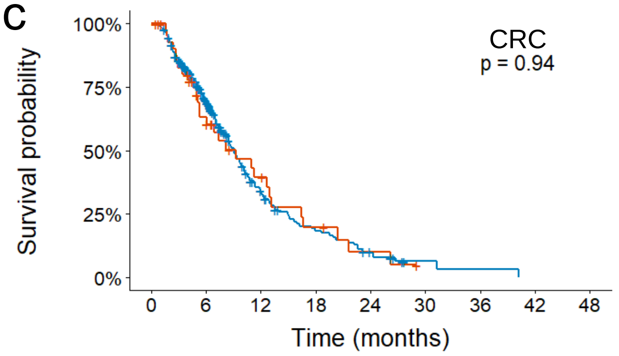

Number at risk

	0	6	12	18	24	30	36	42	48
CH-PD absent	240	124	42	20	9	2	1	0	0
CH-PD present	43	22	11	5	2	0	0	0	0

CRC, 1L	Events/Total	Median PFS (0.95%CI, months)
CH-PD absent	150/240	9.04 (8.19 – 10.3)
CH-PD present	29/43	9.30 (5.33 – 16.4)

d

Colorectal cancer, 1L

	**N**	**Univariate PFS** **HR (95% CI)**	**p-value**
CH-PD	283		
Absent		ref	—
Present		1.02 (0.68, 1.51)	0.94
Diagnosis Age	283	1.01 (0.996, 1.02)	0.18
Smoking History	283		
Never Smoker		ref	—
Current/Former		1.11 (0.83, 1.50)	0.48
Prior RT or Chemotherapy	283		
None		ref	—
Prior RT and Chemotherapy		1.46 (0.82, 2.61)	0.2
Prior Chemotherapy only		1.18 (0.86, 1.62)	0.31
Prior RT only		5.45 (1.32, 22.48)	0.019
MMR status	274		
MSS		ref	—
MSI-H		2.29 (1.49, 3.52)	<0.001
TMB	283	1.008 (1.003, 1.012)	0.002

© 2023 Diplas BH et al. *JAMA Network Open*.

eFigure 4. Univariable Analyses for PFS

a

Esophagogastric cancer, ICB

	Univariate PFS		
	**N**	**HR (95% CI)**	**p-value**
CH-PD	107		
Absent		ref	—
Present		0.92 (0.53, 1.60)	0.76
Diagnosis Age	107	1.00 (0.99, 1.01)	0.82
Smoking History	107		
Never Smoker		ref	—
Current/Former		0.94 (0.63, 1.41)	0.77
Prior RT or Chemotherapy	107		
None		ref	—
Prior RT and Chemotherapy		0.99 (0.55, 1.77)	0.97
Prior Chemotherapy only		1.31 (0.78, 2.20)	0.31
HER2	107		
Negative		ref	—
Positive		1.36 (0.78, 2.36)	0.28
MMR status	96		
MSS		ref	
MSI-H		0.30 (0.14, 0.62)	0.001
TMB	106	0.965 (0.946, 0.984)	<0.001

HR: Hazard ratio; OS: Overall survival; CI: Confidience interval; MMR: Mismatch repair; MSI-H: Microsatellite instability-high; MSS: Microsatellite stable

b

Colorectal cancer, ICB

	Univariate PFS		
	**N**	**HR (95% CI)**	**p-value**
CH-PD	116		
Absent		ref	—
Present		0.83 (0.46, 1.49)	0.53
Diagnosis Age	116	0.99 (0.98, 1.01)	0.3
Smoking History	116		
Never Smoker		ref	—
Current/Former		0.89 (0.60, 1.32	0.55
Prior RT or Chemotherapy	115		
None		ref	—
Prior RT and Chemotherapy		1.03 (0.55, 1.92)	0.94
Prior Chemotherapy only		0.73 (0.42, 1.26)	0.26
MMR status	116		
MSS		ref	—
MSI-H		0.22 (0.13, 0.38)	<0.001
TMB	116	0.975 (0.966, 0.985)	<0.001

© 2023 Diplas BH et al. *JAMA Network Open*.

eFigure 5. No Difference in WBC and Subtype Cell Populations for Metastatic GI Cancer Cohorts Based on CH and CH-PD Status

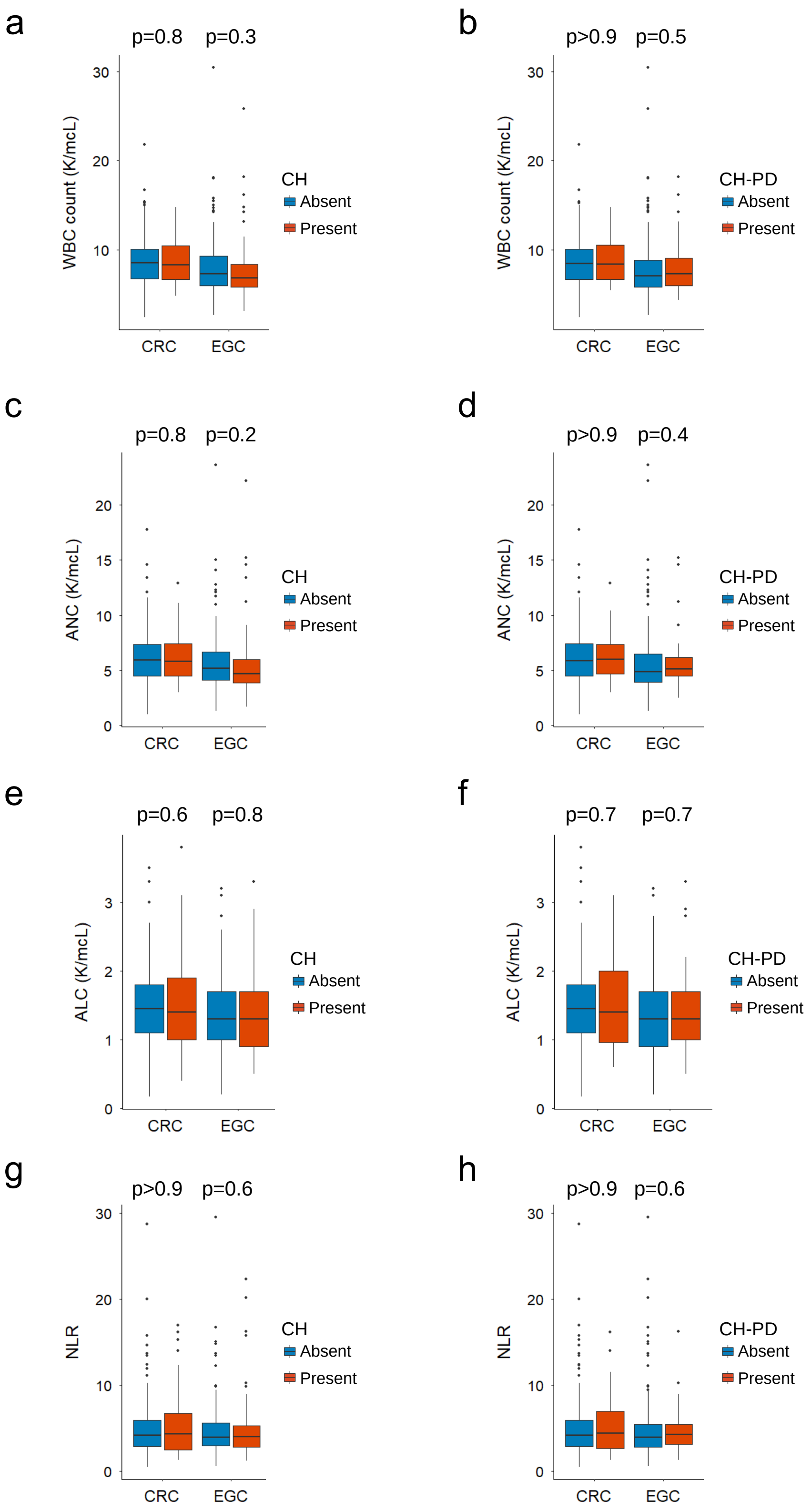

© 2023 Diplas BH et al. *JAMA Network Open*.

eFigure 6. G-CSF Support Based on CH or CH-PD Status

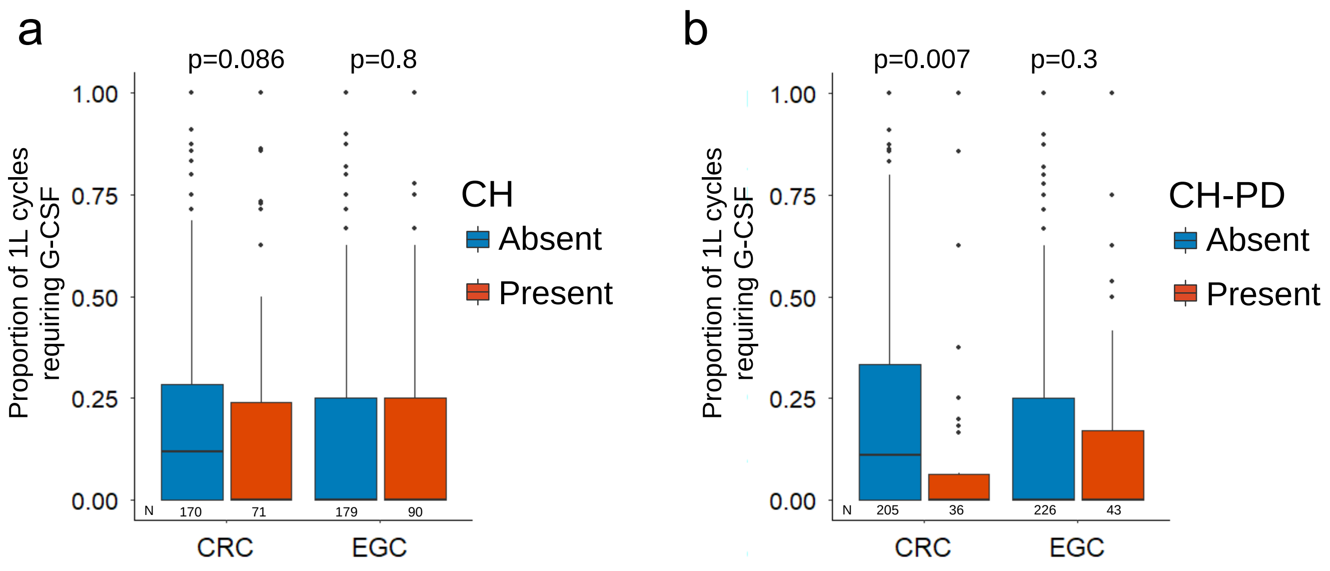

© 2023 Diplas BH et al. *JAMA Network Open*.
