# Supplementary material for: Clinical Importance of Clonal Hematopoiesis in Metastatic Gastrointestinal Tract Cancers
Source: JAMA Netw Open. 2023 Feb 2;6(2):e2254221. doi: 10.1001/jamanetworkopen.2022.54221 (PMC9896303; doi:10.1001/jamanetworkopen.2022.54221)
Supplement: Supplement 2. — Data Sharing Statement [file jamanetwopen-e2254221-s002.pdf]

Data Sharing Statement

Diplas. Clinical Importance of Clonal Hematopoiesis in Metastatic Gastrointestinal Tract Cancers. *JAMA Netw Open*. Published February 02, 2023.
doi:10.1001/jamanetworkopen.2022.54221

Data
Data available: Yes
Data types: Other (please specify)
Additional Information: De-identified additional clinical data can be requested from the corresponding author upon request.
How to access data: CH and CH-PD detection was performed as previously published. CH data are available on https://www.cbioportal.org/study/summary?id=msk_ch_2020.
When available: With publication

Supporting Documents
Document types: Other (please specify)
Additional Information: CH and CH-PD detection was performed as previously published. CH data are available on https://www.cbioportal.org/study/summary?id=msk_ch_2020.
How to access documents: CH and CH-PD detection was performed as previously published. CH data are available on https://www.cbioportal.org/study/summary?id=msk_ch_2020.
When available: With publication

Additional Information
Who can access the data: Additional clinical data can be requested from the corresponding author upon request.
Types of analyses: Additional clinical data can be requested from the corresponding author upon request.
Mechanisms of data availability: Additional clinical data can be requested from the corresponding author upon request.
